# Current Concepts of Non-Coding RNAs in the Pathogenesis of Non-Clear Cell Renal Cell Carcinoma

**DOI:** 10.3390/cancers11101580

**Published:** 2019-10-17

**Authors:** Dominik A. Barth, Ondrej Slaby, Christiane Klec, Jaroslav Juracek, Rares Drula, George A. Calin, Martin Pichler

**Affiliations:** 1 Research Unit of Non-Coding RNAs and Genome Editing, Division of Clinical Oncology, Department of Medicine, Comprehensive Cancer Center Graz, Medical University of Graz, 8036 Graz, Austria; 2 Central European Institute of Technology, Masaryk University, 62500 Brno, Czech Republic; 3 Department of Comprehensive Cancer Care, Masaryk Memorial Cancer Institute, 62500 Brno, Czech Republic; 4 Research Centre for Functional Genomics and Translational Medicine, Iuliu Hatieganu University of Medicine and Pharmacy, 40015 Cluj-Napoca, Romania; 5 Department of Experimental Therapeutics, The University of Texas MD Anderson Cancer Center, Houston, TX 77030, USA

**Keywords:** renal cell carcinoma, non-clear cell, lncRNA, miRNA, long noncoding RNA, microRNA biomarker

## Abstract

Renal cell carcinoma (RCC) is a relatively rare malignancy of the urinary tract system. RCC is a heterogenous disease in terms of underlying histology and its associated underlying pathobiology, prognosis and treatment schedule. The most prevalent histological RCC subtype is clear-cell renal cell carcinoma (ccRCC), accounting for about 70–80% of all RCCs. Though the pathobiology and treatment schedule for ccRCC are well-established, non-ccRCC subtypes account for 20%–30% of RCC altogether, and their underlying molecular biology and treatment options are poorly defined. The class of non-coding RNAs—molecules that are generally not translated into proteins—are new cancer drivers and suppressors in all types of cancer. Of these, small non-coding microRNAs (miRNAs) contribute to carcinogenesis by regulating posttranscriptional gene silencing. Additionally, a growing body of evidence supports the role of long non-coding RNAs (lncRNAs) in cancer development and progression. Most studies on non-coding RNAs in RCC focus on clear-cell histology, and there is a relatively limited number of studies on non-ccRCC subtypes. The aim of this review is to give an overview of the current knowledge regarding the role of non-coding RNAs (including short and long non-coding RNAs) in non-ccRCC and to highlight possible implications as diagnostic, prognostic and predictive biomarkers.

## 1. Introduction

Kidney cancer is the third most common malignancy of the genitourinary system in females and the second most common in males [1]. In the USA, in 2019, an estimated 73,800 patients will be newly diagnosed with, and 14,770 will die from, kidney cancer [1]. The incidence of kidney cancer worldwide accounts for 2.4%, measuring up to 338,000 new cases and 144,000 deaths each year in total [2]. The majority of kidney cancer is diagnosed as renal cell carcinoma (RCC), which accounts for 90% of all renal malignancies [3]. Although a stage migration toward earlier tumor stages occurred in the last two decades [4], cancers of the kidney are responsible for approximately 144,000 deaths annually worldwide [2].

RCC is classified according to its underlying histology, which is defined by distinctive morphological and histological criteria [5]. The most prevalent subtype of RCC is clear-cell renal cell carcinoma (ccRCC), which accounts for 70–80% of all RCC cases. The second and third most common subtypes are papillary (pRCC) and chromophobe (chRCC) renal cell carcinomas, representing 10–15% and 3–5% of RCC, respectively [6]. Other known, but very rare, subtypes include tubulocystic (tcRCC) and clear-cell papillary renal cell carcinoma (ccpRCC) [5]. Several prognostic factors have been identified within recent years including blood-based [7,8] and tissue-based parameters [9,10], which have been summarized in different prognostic scores and prediction tools [11,12,13]. Unfortunately, up to 30% of patients initially present with metastatic disease or develop metastasis within the first two years after diagnosis [14]. Depending on the risk stratification criteria, in this setting, median survival times are 43.2, 22.5 and 7.8 months for good, intermediate and poor risk groups, respectively [15]. Nonetheless, due to the recent introduction of checkpoint inhibitors in the first-line treatment of intermediate or poor risk advanced or metastatic RCC, the prognosis has become more favorable [16]. Yet, this relates only to ccRCC, since the major studies leading to this revolution in RCC-treatment included only patients with clear-cell morphology [17,18,19,20]. As a result, data from large randomized clinical phase III trials on immunotherapy in non-ccRCC are widely missing.

**Figure 1:**
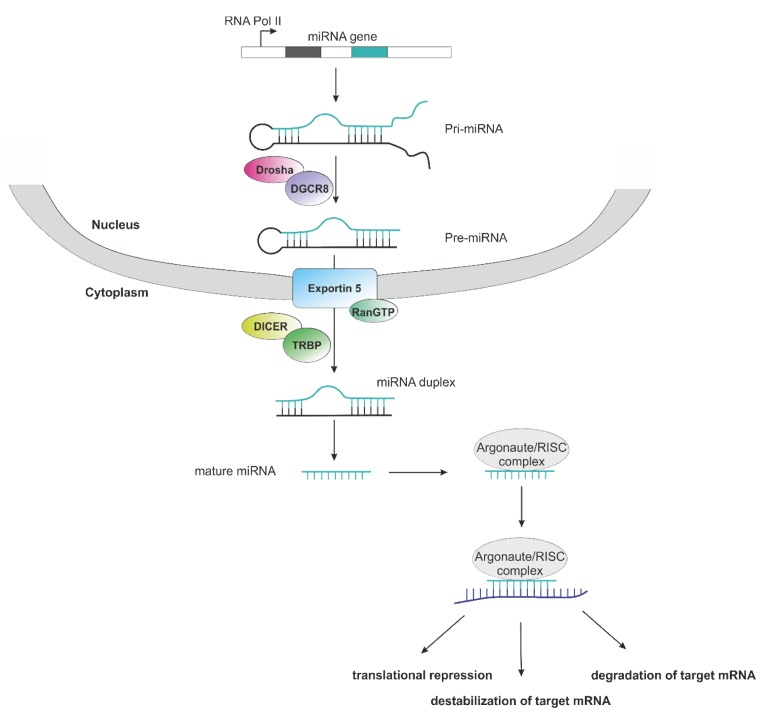
Schematic representation of microRNAs (miRNA) biogenesis and mode of action. Transcription of a miRNA gene is mostly conducted by RNA Polymerase II (pri-miRNA), followed by processing via Drosha/DGCR8 complex into a precursor hairpin miRNA (pre-miRNA). After nuclear export via Exportin 5/RanGTP, the pre-miRNA is processed by Dicer/TRBP into a miRNA duplex without hairpin. One strand of the mature RNA duplex associates with the Argonaut/RISC complex and guides the whole complex to the target mRNA (violet). Consequences of miRNA binding to the mRNA target are translational repression, destabilization or degradation of the target mRNA.

One major reason for the lack of clinical trials in non-clear cell RCC is its heterogenous underlying biology. This also conveys to the emerging field of non-coding RNA in cancer, where a high number of studies reported on the impact of microRNAs (miRNAs) on cancer development, progression and potential clinical implications in ccRCC. Data on miRNAs and long non-coding RNAs (lncRNAs) in non-clear cell RCC remains limited. In general, miRNAs represent small RNA molecules composed of about 19–25 nucleotides, which are not coding for proteins. They contribute to the regulation of posttranscriptional gene silencing as they bind to the 3′-untranslated region (3′-UTR) of the targeted messenger RNA of the respective target gene, thus leading to its degradation or destabilization and consequently, inhibited translation [21] (Figure 1). This happens due to the miRNA’s association with Argonaute (Ago) proteins. Together, they form the RNA-induced silencing complex (RISC) [21].

A single miRNA may target more than 100 possible different sites [22]. The impact of miRNAs in cancer has been investigated in various cancer entities, such as colorectal cancer [23,24,25], testicular cancer [26,27], B-cell lymphoma [28], breast cancer [29], glioma [30,31,32], nephroblastoma [33], pancreatic cancer [34] or leukemia [35,36]. miRNAs were said to contribute to cancer development, progression or even drug resistance, and may be used as future biomarkers or in cancer therapy [37,38].

**Figure 2:**
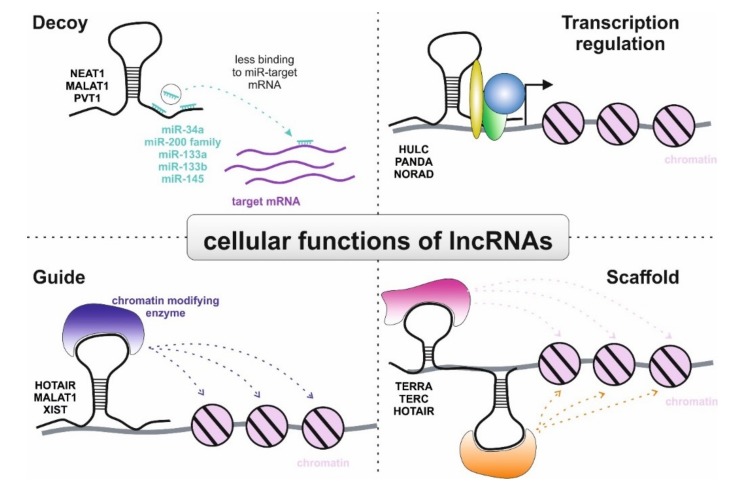
Graphical overview of cellular lncRNA actions. Decoy: long non-coding RNAs (lncRNA) can act as molecular sponges for miRNAs and transcription factors (not depicted), thereby preventing these molecules from binding to their actual targets. Transcription regulation: in combination with diverse transcription factors, lncRNAs possess the ability to regulate cellular transcription. Guide: lncRNA can act as guides for chromatin-modifying enzymes to target genes. Scaffold: lncRNAs are able to form ribonucleoprotein complexes with different protein partners. This complex, consisting of lncRNAs and proteins can regulate transcription. In each panel, representative examples of lncRNAs are given.

In contrast to the shorter miRNAs, lncRNAs are defined as longer than 200 nucleotides in length and are commonly located in the nucleus of a cell [39,40]. In recent years, different functions of lncRNAs have been demonstrated (Figure 2). lncRNAs may act as a decoy for other molecules [41], for instance, miRNAs, thus leading to less translational repression, degradation or destabilization of the target mRNAs [42]. Further mechanisms include guide [43] and scaffold [44] lncRNAs and the involvement in transcription regulation [39,42]. In cancer, lncRNAs were shown to influence gene transcription, as well as cell cycle regulation and proliferation, thus representing potential therapeutic targets [45,46].

The present review aims to summarize the current literature of non-coding RNA research in five non-clear cell RCC histologies and to focus on their potential clinical implementations in diagnosis, prognosis and therapy.

## 2. Methods

The literature research was conducted using the PUBMED database. Various combinations of the search terms “renal cell carcinoma”, “kidney cancer”, “papillary”, “chromophobe”, “tubulocystic”, “clear cell papillary”, “non-clear cell”, “long non coding RNA”, “lncRNA”, “microRNA”, “miRNA” or “non-coding RNA” were used and article headlines, abstracts and full texts were screened for relevance on the topic. Studies that investigated non-coding RNAs in at least one non-clear cell RCC subtype were included in this review.

## 3. Discussion

### 3.1. Role of microRNAs in Non-Clear Cell RCC

#### 3.1.1. Papillary Renal Cell Carcinoma

With a prevalence of about 10% of all RCC cases, pRCC is the second most common histological subtype of RCC [47] and can be differentiated into two different subsets by distinctive histological and molecular findings [48]. Both type 1 and type 2 are characterized by mostly papillary and tubular structures. However, in type 1, these respective structures are predominantly covered by small cells with pale cytoplasm and small oval nuclei, whereas in type 2, they are covered by larger eosinophilic cells with large spherical nuclei [49]. A large proportion of type 1 tumors have multiple chromosomal alterations, the most frequent of which are gains of chromosomes 7 and 17. In addition, MET mutations are common in type 1 tumors. In type 2, CDKN2A alterations, either by mutations or by hypermethylation, occur frequently [50]. Overall, patients with pRCC have a better survival outcome than those with ccRCC; however, type 2 tumors are usually more aggressive and have a greater metastatic potential [9]. Therefore, type 2 pRCC has a poorer prognosis than type 1 papillary and even ccRCC [51,52].

##### Diagnostic Potential of miRNAs in pRCC

The number of miRNAs with potential for diagnostic purposes is described in this paragraph. miR-21 is upregulated in malignant renal tumors compared to healthy renal tissue [53,54] and is linked to tumor growth, cancer progression and metastases [55]. Interestingly, different levels of miR-21 expression have been found among different renal tumor subtypes. There is a significantly higher expression of miR-21 in clear-cell and papillary subtypes, in contrast with chromophobe RCC and oncocytoma [53,56]. In addition, in pRCC, increased miR-21 expression (gene locus 17q23.1) is linked to copy number changes of the genome, since pRCC cells feature a high frequency of trisomy 17 and, therefore, an increase of the related gene products [56]. As mentioned earlier, numerical chromosomal alterations are more frequently associated with type 1 pRCC [50]. Thus, miR-21 could be used to distinguish between these respective RCC subtypes on a molecular basis with relatively high sensitivity (83%) and specificity (90%); however, a differentiation of ccRCC and pRCC cannot be achieved by using miR-21 only, meaning that molecular diagnostics are not a substitute for an experienced pathologist [53].

On the other hand, Powers and colleagues [56] identified 3 miRNAs with distinctive levels of expression in ccRCC and pRCC. miR-126, miR-126* and miR-143 were significantly upregulated in ccRCC compared to pRCC, which made it possible to distinguish between the respective two RCC subtypes [56].

The downregulation of miR-126 in pRCC relative to ccRCC was confirmed by another study that also aimed to correctly discriminate between different RCC subtypes [57]. A two-step model for differentiating ccRCC and pRCC from chRCC and oncocytoma was proposed by Di Meo et al. [57] based on the expression rates of miR-221, miR-222 and miR-126. The first step takes into consideration the differential expression rates of either miR-221 or miR-222. Both, miR-221 and miR-222 display decreased expression levels in carcinomas with clear-cell or papillary morphology. The second step involves discriminating between ccRCC and pRCC based on the expression rate of miR-126, which is increased in ccRCC compared to pRCC [57]. The location of miR-126 on chromosome 9 (gene locus 9p34.3) and the frequent loss of chromosome 9p characteristic for type 2 pRCC might be a feasible explanation for the lower expression compared to ccRCC [50,57]. Regarding the role of miR-126 in carcinogenesis, miR-126 targets the 3′-UTR of vascular endothelial growth factor A (VEGF-A) [58] and additionally, expression levels correlate inversely with the expression of epidermal growth factor-like domain 7 (EGFL7) [59]. Both, VEGF-A and EGFL7 are involved in tumor angiogenesis [58,59]. Furthermore, miR-126 may be involved in the regulation of the PI3K/Akt pathway [60].

Wach and colleagues [61] conceived a study in which, besides the discrimination of both healthy and cancer tissue, as well as ccRCC and pRCC subtypes, they were also able to differentiate type 1 and 2 of pRCC by using a multistep combination of miRNAs. In the first step, the miRNAs used to distinguish between healthy and tumor tissue were miR-145, miR-200c, miR-210 and mi-R502-3p. In the second step, miR-145 and miR-503-3p were used to classify ccRCC versus pRCC, whereas in the third step, type 1 and 2 were distinguished by utilizing miR-210 and miRNA let-7c. Both, miR-210 and let-7c were upregulated in type 1 pRCC as compared with type 2. The subtypes were classified correctly in 86.5%, 77.6% and 86.4% for the first, second and third discrimination, respectively [61].

miR-210 is linked to hypoxia in cancer tissue and is directly involved in the hypoxia pathway. Hypoxia-induced factor 1 alpha (HIF1α) binds to its promotor region, thus inducing the transcription of miR-210 in a proposed positive feedback loop [62,63]. Nonetheless, higher HIF1α levels and, consequently, elevated miR-210 expression may also occur in non-hypoxic states due to mutations of the Von Hippel Lindau (VHL) gene, which leads to insufficient HIF1α-degradation [64]. To explain the relatively low miR-210 expression in type 2 pRCC, Wach et al. suggest less dependence on hypoxia in type 2 compared to type 1 pRCC [61]. Furthermore, miR-210 is upregulated in various other cancer entities, including ccRCC [62,65], and has been introduced as a possible diagnostic and prognostic biomarker in RCC, as well as for other malignancies, such as colorectal cancer [66,67].

Targets of the let-7 family include the oncogenes RAS and MYC, making let-7 family members veritable tumor-suppressing miRNAs. The relative downregulation of let-7c in type 2 pRCC corroborates the recent discovery of MYC overexpression in the respective subtype [61,68]. In addition, immunohistochemical MYC straining patterns could discriminate prognostic groups in type 1 pRCC [69].

Regarding the distinction of RCC subtypes, miR-155 showed higher expression levels in ccRCC compared to pRCC and, therefore, could be useful in the distinction of the two subtypes [54]. Moreover, miR-155 also carries prognostic information, as its overexpression is related to decreased disease-specific survival (DSS) in RCC, although this only prevailed in the univariate analysis [54]. miR-155 is located on chromosome 21 (gene locus 21q21.2–21.3) and is linked to tumor proliferation. It directly targets nedd4-family interacting protein 1 (NDFIP1), which is a part of the regulation of PTEN (Phosphatase and tensin homolog) [70], a commonly known apoptosis-promoting tumor suppressor gene in various solid malignancies that is also associated with poorer survival in kidney cancer [71]. miR-155 may target the 3′-UTR of PTEN mRNA directly as well, leading to an activation of the PI3K/Akt pathway and thus, promoting tumor progression [72]. Other functions of miR-155 that are related to carcinogenesis are targeting the tumor suppressor DMTF1 (Cyclin D Binding Myb-Like Transcription Factor 1) and enhancing the Wnt/beta-catenin pathway [31,73].

##### Prognostic Potential of miRNAs in pRCC

miRNAs are not only useful as potential diagnostic biomarkers but have also proven to be of prognostic significance in various cancer entities, such as breast, gastric, colon and prostate cancer [74,75,76,77]. Other than the previously discussed studies, which mainly addressed the potential of microRNAs in diagnosis and the classification of RCC, some studies also focused on their possible prognostic value in pRCC (Table 1).

Decreased expression levels of miR-200c and miR-127, as well as high levels of miR-34a, were associated with better overall survival (OS) in patients with pRCC [78]. However, only miR-34a proved to be an independent prognostic marker in the multivariate analysis in the validation stage [78]. miR-200c was shown to be dysregulated in many solid tumor entities, such as, but not limited to, bladder, breast, colorectal, gastric and lung cancer [82]. Moreover, it is involved in the proliferation and differentiation of normal and cancer stem cells and, by modifying the cellular sensitivity to death receptor CD95, in the regulation of apoptosis. miR-200c may also suppress endothelial-to-mesenchymal transmission (EMT) and, therefore, inhibit tumor progression [82].

miR-34a may function as a tumor suppressor, which could illuminate why higher expression levels favor a better prognosis. E2F3, MET and Fra-1 are associated targets of miR-34a [83,84,85].

Other miRNAs analyzed in respect of their prognostic potential are hsa-miR-1293 and hsa-miR-3199 2 [79]. In the study, the cut-off was chosen as the median expression level. A significant difference in progression-free survival (PFS) in a 5-year follow-up for high-risk (39.4%) and low-risk (70.3%) groups was reported [79]. However, the inclusion of both metastasized and non-metastasized patients in the analysis must be noted as a limitation of the study regarding the calculation of PFS. Except for hsa-miR-1193, which was also shown to relate to lung cancer [86], to date, there are no studies to validate their involvement in carcinogenesis and tumor progression.

In a competing endogenous RNA (ceRNA) network analysis, seven miRNAs have been found that may be promising candidates as prognostic biomarkers in pRCC [80]. Higher expression levels of hsa-miR-133a, hsa-miR-133b, hsa-miR-145, hsa-miR-216a, hsa-miR-217 and hsa-miR-1297 were associated with detrimental effects on OS. As opposed to this, an increased expression of hsa-miR-211 was connected to a better OS [80]. However, since no uni- and multivariate models were used in the survival analysis, there is not enough evidence to support the prognostic impact of the respective miRNAs, yet. As for miR-145, at least in the previously mentioned study of Wach et al. [61], it did not independently predict prognosis, as it failed to reach statistical significance in multivariate models.

#### 3.1.2. Chromophobe Renal Cell Carcinoma

chRCC is the third most common subtype of RCC and accounts for 5% of this cancer entity [47,87]. Metastatic disease at diagnosis is less common in patients with chRCC than with ccRCC and altogether, chRCC has more favorable survival rates. However, this is not true for advanced metastatic chRCC, which shows a less favorable outcome [88].

The diagnosis of chRCC by morphologic characteristics alone can be difficult, since the eosinophilic variant of ccRCC, and especially oncocytoma, are possible differential diagnoses, which all show oncocytic histological features [89].

##### Diagnostic Potential of miRNAs in chRCC

As shown previously, the use of miRNAs could facilitate the correct subtyping of RCC, including chRCC. Multiple studies have already investigated the diagnostic potential of miRNAs in chRCC, focusing especially on the discrimination of chRCC and oncocytoma [56,57,90,91,92].

miR-221 and miR-222 are consistently overexpressed in both chRCC and oncocytoma [56,57,90,91,93]. Altered expression patterns have already been demonstrated in multiple other solid malignancies, including glioma, colorectal, gastric, breast and prostate cancer [32,94,95,96,97]. In ccRCC, targeting TIMP2 (Tissue inhibitor of metalloproteinases 2), miR-221 was reported to enhance proliferation, migration and invasion [98]. Moreover, high expressions of both, miR-221 and miR-222 are associated with poorer treatment response in patients with metastatic renal cell carcinoma treated with the multikinase inhibitor sunitinib. Potential targets of miR-221/222 are VEGFR2 and c-KIT, which are both targets of sunitinib [99].

miR-203 is upregulated in chRCC relative to oncocytoma [90]. Conversely, it is downregulated in ccRCC, which was associated with worse outcome. Also, transient forced expression resulted in the inhibition of further proliferation and metastatic spread, which indicates the tumor suppressing role of miR-203 in renal neoplasms. miR-203 directly targets fibroblast growth factor 2 (FGF2) [100].

Powers et al. [56] found miR-429, miR-200b, miR-629* and miR-124 to be significantly differentially expressed between chRCC and oncocytoma and relatively overexpressed in chRCC, respectively. In addition, miR-200c was increased in chRCC.

miR-200b/c and miR-429 are members of the miR-200 family, which targets the EMT-activating transcription factors ZEB1 and ZEB2. As a result, the miR-200 family is connected to the regulation of EMT [101]. EMT, a hallmark of cancer, is an important step in the disease progression, as it comes with increased invasive and metastatic potential of cancer cells [102].

The distinctive miRNA characteristics of chRCC were used in a tested two-step model for the differentiation of RCC subtypes. miR-221 and miR-222 could correctly distinguish ccRCC and pRCC from chRCC and oncocytoma, respectively, since they show inverse levels of expression. In a second step, regarding the subclassification of tumors, miR-200b, miR-200c and miR-222 could significantly distinguish chRCC form oncocytoma. Interestingly, miR-200b alone showed greater discriminatory ability than various combinations among these miRNAs and could differentiate chRCC and oncocytoma with a sensitivity and specificity of over 89% and 90%, respectively [57].

Another study of Fridman et al. [92] also used a two-step approach to identify RCC subtypes and oncocytomas. Expression levels of miR-221 and miR-210 differentiated the pairs of chRCC and oncocytoma versus ccRCC and pRCC, whereas miR-200c and miR-139-5p were used to identify chRCC or oncocytoma. 

##### Prognostic Potential of miRNAs in chRCC

Pretreatment expression levels of specific miRNAs may also qualify as prognostic biomarkers in chRCC. miR-191, miR-19a, miR-210 and miR-425 were associated with recurrence-free survival (RFS) in a retrospective study including 58 patients with primary chRCC. However, only miR-210 proved to be an independent biomarker for RFS in the uni- and multivariate Cox analysis. Moreover, high expression of the respective miRNAs and additionally, of miR-186 was associated with shorter OS, yet this did not prevail in the uni- and multivariate analysis. Notably, the relatively short median follow-up of 63 months, at a 5-year survival of 85% in chRCC may contribute to the insignificant results [81]. Nonetheless, the results of this study are in line with previous studies, which found overexpression of miR-210 in adverse prognosis of ccRCC [103].

#### 3.1.3. Tubulocystic Renal Cell Carcinoma

tcRCC is an extremely rare histological subtype of RCC, that is often an incidental finding of radiological evaluation of Bosniak type III and IV cysts of the kidney [104]. As the tumor is indolent in most cases, patients usually present themselves asymptomatic at diagnosis and rarely metastasized [105]. Macroscopically, it features multiple cysts varying in size. Histologically, it shows enlarged nucleoli and an eosinophilic cytoplasm similar to oncocytomas [5]. Therefore, the differentiation of tcRCC from other RCC subtypes and oncocytomas may be difficult [106]. However, molecular profiling, as well as a unique miRNA expression profile may facilitate diagnosis of tcRCC.

##### miRNA Expression in tcRCC

One study focused on tcRCC and compared miRNA expression levels with other RCC subtypes, namely ccRCC, pRCC and ccpRCC [107]. miR-138, miR-200c and miR-182 were found to be upregulated in tcRCC compared to the other subtypes; however, the difference for miR-182 was not significant [107].

Additionally, downregulated expressions of miR-210, miR-34a and miR-155 were noted; however, only the expression levels of miR-155 were significantly decreased compared to both ccRCC and pRCC, whereas miR-210 and miR-34a were significantly downregulated compared to both ccRCC or pRCC [107]. The roles of miR-155 and miR-210 in cancer have already been discussed. As for miR-34a, it is closely related to p53, an important and commonly known tumor suppressor, which is frequently mutated across multiple cancer entities. It transcriptionally controls the expression of miR-34a [108]. miR-34a itself is seen as a tumor suppressor, as its targets are widely associated with proliferation processes, apoptosis, cell aging, cancer stem-like cell phenotype, EMT, cell motility and even immune evasion [108]. Furthermore, miR-34a is upregulated in ccRCC [109]. Amongst others, promising targets are MYC, NOTCH1, MET, BCL2, CD44 or PD-L1 [108]. As a result, a phase I trial testing a liposomal miR-34a mimic has been conducted, for possible implementation in the future treatment of cancer [110].

In the study by Lawrie et al. [107], no alteration of miRNA expression was distinctive for tcRCC only. Nonetheless, a combination of different miRNAs may be used to discriminate tcRCC from other RCC subtypes in future studies.

#### 3.1.4. Clear-cell Papillary Renal Cell Carcinoma

ccpRCC is a rare, mostly indolent subtype of renal cancer that accounts for up to 5% of resected renal tumors [111]. Higher incidence is seen in patients with end-stage renal disease or chronic kidney disease [112]. Histologically, it shows low-grade clear epithelial cells in a tubular and papillary arrangement [5].

##### miRNA Expression in ccpRCC

miR-200a, miR-200b, miR-200c, miR-141 and miR-429, which are all part of the miR-200 family, are consistently upregulated in ccpRCC. However, they are downregulated in other renal carcinoma subtypes, including clear-cell and papillary morphology subsets [113,114].

As the miR-200 family is linked to the regulation of EMT [115], Lawrie et al. [113] further show that, conversely to renal neoplasms with clear-cell morphology in ccpRCC, the markers vimentin (mesenchymal), E-cadherin (epithelial) and β-catenin (mesenchymal) were all positive in immunofluorescent staining. Due to this unique characteristic, indicating incomplete EMT in ccpRCC, the authors suggest a potential use in differential diagnosis of RCC subtypes [113].

Furthermore, ccpRCC shows high levels of miR-210 expression [113,114]. As previously stated, miR-210 is involved in the hypoxia pathway [62,113,114], which activation has already been shown in ccpRCC. However, this activation is independent of the usual hypoxia pathway-linked functional loss of the Von Hippel Lindau (VHL) gene, which is still functional in ccpRCC as opposed to ccRCC [114,116]. Respectively, Lawrie et al. [113] suggest epigenetic regulation as a possible explanation, as they found two specific miRNAs (miR-885 and miR-378b), located downstream and close to the VHL gene, being upregulated in ccpRCC. Additional miRNAs found to be overexpressed in ccpRCC are miR-34a, miR-182, miR-424, miR-122, miR-21, miR-34b*, miR-489 [113,114]. miR-122 might be connected to the regulation of VHL and, therefore, also be involved in the hypoxia pathway [117]. In addition, it contributes to proliferation, invasion and an increased metastatic potential in ccRCC [118,119,120].

Downregulated miRNAs in ccpRCC are miR-4284, miR-1202, miR-135a, miR-1973 and miR-204 [114]. Interestingly, patterns of upregulated miRNAs expression are highly similar in ccpRCC and ccRCC, whereas the same applies for ccpRCC and pRCC in terms of downregulated miRNA expression [114].

#### 3.1.5. Xp11 Translocation Renal Cell Carcinoma

Xp11 translocation RCC is the most common renal neoplasm in children. However, it is also seen in adults, where its prevalence may be underestimated. Xp11 RCCs show characteristic chromosomal translocations at a breaking point—the locus Xp11.2—resulting in a distinctive gene fusion including the TFE3 transcription factor gene. Microscopically, this results in a tumor showing clear cells, as well as papillary structures [121].

##### miRNA Expression in Xp11 RCC

In a genome-wide miRNA expression profile analysis, the expression levels of several miRNAs were found to be specifically altered in Xp11 RCC relative to other malignancies of the kidney, such as ccRCC, pRCC and ccpRCC. Compared to healthy renal tissue, miR-148a-3p, miR-221 3p, miR-185-5p, miR 196b-5p and miR-642a-5p are upregulated, whereas miR-133b and miR-658 were downregulated [122].

Regarding the overall miRNA expression profiles, they were most similar to ccpRCC and showed the biggest difference to pRCC, respectively [122].

### 3.2. Role of lncRNAs in Non-Clear Cell RCC

#### 3.2.1. Papillary Renal Cell Carcinoma

Mutations of the putative promoter region of the lncRNA NEAT1 (nuclear-enriched abundant transcript 1) were found in 17% of pRCC. This came along with an overexpression of NEAT1 and is associated with decreased survival. Additionally, lncRNA MALAT1 (metastasis-associated lung adenocarcinoma transcript 1) was co-expressed with NEAT1 in pRCC, as well as in ccRCC [123]. NEAT1 is frequently overexpressed in cancer tissues, including ccRCC, where it is linked to EMT and chemoresistance by competing with miR-34a and thus, influencing the miR-34a/c Met axis [124,125]. Mounting evidence suggests the role of NEAT1 as a competitive endogenous RNA and proposes its potential use as a biomarker and therapeutic target in cancer [124]. MALAT1 was shown to influence tumor progression and prognosis in ccRCC, as well as acting as a competing endogenous RNA by sponging the miRNA-200 family [126,127] (Figure 2).

In a ceRNA network analysis using the TCGA (The Cancer Genome Atlas) database, lncRNA PVT1 (Plasmacytoma Variant Translocation 1) was suggested to compete with three miRNAs, namely miR-133a, miR-133b and miR-145, thus regulating the expression of the SP1 gene. SP1 is a transcription factor involved in many tumorigenesis-associated processes and was identified as a potential target of the respective miRNAs. In the study, an increased expression of PVT1 was associated with shorter OS. Furthermore, high levels of lncRNAs AC003092.1, RP6-191P20.4, RP11-401P9.4 and RP11-496D24.2 correlated with worse prognosis [80]. However, these results should only be viewed as hypothesis-generating, since uni- and multivariate regression models were not used in the survival analysis.

Luo et al. [128], too, conducted a ceRNA network analysis using the TCGA database and found lncRNA MEG3 (maternally expressed 3) to be significantly downregulated in pRCC compared to healthy kidney tissue. MEG3 was suggested to be an important regulator in the network, as it was shown to potentially interact with 14 differentially expressed miRNAs in pRCC, more than any other lncRNA in the study [128]. The role of MEG3 as a tumor suppressor was shown in various cancers such as pancreatic, breast, esophageal and gastric cancer [129,130,131,132]. In ccRCC, MEG3 expression was shown to be downregulated, resulting in decreased apoptosis and accelerated proliferation, migration and invasion, due to its regulatory influence on miR-7/RASL11B signaling [133]. Furthermore, in the present study on pRCC, MEG3 was identified as part of a subnetwork together with lncRNA PWRN1 (Prader-Willi Region Non-Protein Coding RNA 1), miR-508 and miR-21 [128].

lncRNAs may be promising prognostic biomarkers in pRCC (Table 2).

Lan and colleagues [134] created a prognostic index including seven differentially expressed lncRNAs that each independently predicted for cumulative survival. High expression of lncRNA AFAP1-AS1, as well as low expressions of lncRNAs GAS6-AS1, RP11-1C8.7, RP11-21L19.1, RP11-503C24.1, RP11536I6.2 and RP11-63A11.1, were significantly associated with worse prognosis. The calculated prognostic index combining the prognostic value of these lncRNAs could independently differentiate between high- and low-risk groups with an average survival of 109.4 vs. 117.3 months, respectively. The prognostic index also predicted for clinicopathological stages, such as metastasis, nodal invasion and tumor stages [134]. However, the analysis was carried out within the TCGA database and needs further experiments and verification.

Another approach to combine the prognostic value of individual differentially expressed lncRNAs in three subtypes of RCC was suggested by Zuo and colleagues [135]. A prognostic signature of six lncRNAs, independently predicting for OS in all three, ccRCC, pRCC and chRCC, respectively, was established. The lncRNAs included in the signature are AC003092.1, AC079160.1, COL18A1-AS1, LINC00520, LINC02154 and SLC7A11-AS1. All lncRNAs were upregulated in the tumor tissue, compared to healthy renal tissue, and upregulated in high-risk vs low-risk groups, except for COL18A1-AS1, which showed converse behavior. The six lncRNA signatures could also distinguish good and poor prognostic groups within independent prognostic factors such as age, stage III and stage IV [135]. This study, too, was conducted using the TCGA database.

#### 3.2.2. Chromophobe Renal Cell Carcinoma

Data on lncRNA in chRCC is limited. Seven lncRNAs were reported to be associated with prognosis and, therefore, may be used as future biomarkers in chRCC. He et al. [136] performed a ceRNA network analysis and described 16 lncRNAs and 18 miRNAs that might interact with each other, influencing 12 cancer-related pathways.

Moreover, low levels of the six lncRNAs COL18A1-AS1, BRE-AS1, SNHG7, TMEM51-AS1, C21orf62-AS1 and LINC00336 and high expression of LINC00882 significantly predicted for shorter OS [136]. However, multivariate regression models were not used in the analysis. Additionally, the patient cohort extracted from the TCGA database must be noted as a limitation and the results warrant verification in other cohorts and by biological models [136].

## 4. Conclusions

Important differences in miRNA expression patterns among non-clear cell RCC subtypes have been demonstrated. Also, their potential use in diagnostics and differential diagnosis has been suggested; however, further research is warranted to find the most suitable miRNAs for future implementation in clinical practice. Moreover, besides their potential diagnostic capabilities, miRNA expression levels convey prognostic and even predictive information in some non-clear cell RCC subtypes and may influence clinical decision making.

As for lncRNA, data on non-ccRCC is limited and only available for two distinctive subtypes. In papillary and chromophobe RCC, lncRNAs may represent novel prognostic biomarkers and add further insight into the pathogenesis of RCC.

## Figures and Tables

**Table 1:** miRNAs significantly associated with outcomes in non-ccRCC.

Reference	miRNA	Expression Level	Endpoint	Outcome	Independent in Multivariate Analysis
**papillary RCC**					
Ge et al., 2015 [78]	miR-200c	↓	OS	good	no
	miR-127	↓	OS	good	no
	miR-34a	↑	OS	good	yes
Luo et al., 2017 [79]	hsa-miR-1293	↑	PFS	poor	N/E
	hsa-miR-3199-2	↑	PFS	poor	N/E
Huang et al., 2017 [80]	miR-133a	↑	OS	poor	N/E
	miR-133b	↑	OS	poor	N/E
	miR-145	↑	OS	poor	no
	miR-216a	↑	OS	poor	N/E
	miR-217	↑	OS	poor	N/E
	miR-1297	↑	OS	poor	N/E
	miR-211	↑	OS	good	N/E
**chromophobe RCC**					
Ge et al., 2015 [81]	miR-191	↑	RFS, OS	poor	no
	miR-19a	↑	RFS, OS	poor	no
	miR-210	↑	RFS, OS	poor	yes (RFS)
	miR-425	↑	RFS, OS	poor	no
	miR-186	↑	OS	poor	no

Abbreviations: RCC—renal cell carcinoma; OS—overall survival; PFS—progression-free survival; RFS—recurrence-free survival; N/E—not evaluated, no multivariate analysis conducted.

**Table 2:** lncRNAs significantly associated with outcomes in non-ccRCC.

Reference	lncRNA	Expression Level	Endpoint	Outcome	Independent in Multivariate Analysis
**PapillaryRCC**					
Lan et al., 2017 [134]	AFAP1-AS1	↑	OS	poor	N/E *
	GAS6-AS1	↓	OS	poor	N/E *
	RP11-1C8.7	↓	OS	poor	N/E *
	RP11-21L19.1	↓	OS	poor	N/E *
	RP11-503C24.1	↓	OS	poor	N/E *
	RP11536I6.2	↓	OS	poor	N/E *
	RP11-63A11.1	↓	OS	poor	N/E *
Zuo et al., 2018 [135]	AC003092.1	↑	OS	poor	N/E *
	AC079160.1	↑	OS	poor	N/E *
	COL18A1-AS1	↓	OS	poor	N/E *
	LINC00520	↑	OS	poor	N/E *
	LINC02154	↑	OS	poor	N/E *
	SLC7A11-AS1	↑	OS	poor	N/E *
**chromophobe RCC**					
He et al., 2016 [136]	COL18A1-AS1	↓	OS	Poor	N/E
	BRE-AS1	↓	OS	Poor	N/E
	SNHG7	↓	OS	Poor	N/E
	TMEM51-AS1	↓	OS	Poor	N/E
	C21orf62-AS1	↓	OS	Poor	N/E
	LINC00336	↓	OS	Poor	N/E
	LINC00882	↑	OS	poor	N/E

Abbreviations: RCC—renal cell carcinoma; OS—overall survival; N/E—not evaluated; no multivariate analysis conducted. * Combined prognostic indices proved as independent biomarkers.
